# Comparing the performance of screening surveys versus predictive models in identifying patients in need of health-related social need services in the emergency department

**DOI:** 10.1371/journal.pone.0312193

**Published:** 2024-11-20

**Authors:** Olena Mazurenko, Adam T. Hirsh, Christopher A. Harle, Joanna Shen, Cassidy McNamee, Joshua R. Vest

**Affiliations:** 1 Department of Health Policy & Management, Indiana University Richard M. Fairbanks School of Public Health–Indianapolis, Indianapolis, Indiana, United States of America; 2 Regenstrief Institute, Indianapolis, Indiana, United States of America; 3 School of Science, Indiana University–Indianapolis, Indianapolis, Indiana, United States of America; 4 Department of Biostatistics, Harvard T.H. Chan School of Public Health, Boston, Massachusetts, United States of America; Florida State University, UNITED STATES OF AMERICA

## Abstract

**Background:**

Health-related social needs (HRSNs), such as housing instability, food insecurity, and financial strain, are increasingly prevalent among patients. Healthcare organizations must first correctly identify patients with HRSNs to refer them to appropriate services or offer resources to address their HRSNs. Yet, current identification methods are suboptimal, inconsistently applied, and cost prohibitive. Machine learning (ML) predictive modeling applied to existing data sources may be a solution to systematically and effectively identify patients with HRSNs. The performance of ML predictive models using data from electronic health records (EHRs) and other sources has not been compared to other methods of identifying patients needing HRSN services.

**Methods:**

A screening questionnaire that included housing instability, food insecurity, transportation barriers, legal issues, and financial strain was administered to adult ED patients at a large safety-net hospital in the mid-Western United States (n = 1,101). We identified those patients likely in need of HRSN-related services within the next 30 days using positive indications from referrals, encounters, scheduling data, orders, or clinical notes. We built an XGBoost classification algorithm using responses from the screening questionnaire to predict HRSN needs (screening questionnaire model). Additionally, we extracted features from the past 12 months of existing EHR, administrative, and health information exchange data for the survey respondents. We built ML predictive models with these EHR data using XGBoost (ML EHR model). Out of concerns of potential bias, we built both the screening question model and the ML EHR model with and without demographic features. Models were assessed on the validation set using sensitivity, specificity, and Area Under the Curve (AUC) values. Models were compared using the Delong test.

**Results:**

Almost half (41%) of the patients had a positive indicator for a likely HRSN service need within the next 30 days, as identified through referrals, encounters, scheduling data, orders, or clinical notes. The screening question model had suboptimal performance, with an AUC = 0.580 (95%CI = 0.546, 0.611). Including gender and age resulted in higher performance in the screening question model (AUC = 0.640; 95%CI = 0.609, 0.672). The ML EHR models had higher performance. Without including age and gender, the ML EHR model had an AUC = 0.765 (95%CI = 0.737, 0.792). Adding age and gender did not improve the model (AUC = 0.722; 95%CI = 0.744, 0.800). The screening questionnaire models indicated bias with the highest performance for White non-Hispanic patients. The performance of the ML EHR-based model also differed by race and ethnicity.

**Conclusion:**

ML predictive models leveraging several robust EHR data sources outperformed models using screening questions only. Nevertheless, all models indicated biases. Additional work is needed to design predictive models for effectively identifying all patients with HRSNs.

## Introduction

Healthcare organizations in the United States face growing expectations to address patients’ health-related social needs (HRSNs) as issues such as housing instability, food insecurity, and financial strain affect health, well-being, and healthcare costs [1]. Patients with HRSNs require referrals to social service organizations, connections with social workers, or access to relevant resources to meet their specific HRSNs, like food or transportation vouchers [2]. Interventions to address HRSNs appear to be a promising solution to reducing unnecessary healthcare utilization and improving health [3].

Healthcare organizations must first correctly identify patients with HRSNs to refer them to appropriate services or offer resources to address their HRSNs [4,5]. This has proven challenging [6]. For one, identifying patients with HRSNs requires changes to clinical workflows by increasing clinician and patient data collection burdens and changes to information technology systems to record and ultimately leverage HRSN information [6]. Additionally, even successful interventions to identify patients with HRSNs will leave some patients unscreened due to non-responses, language barriers, breakdowns in workflow, time constraints, or uncertainty over screening responsibilities [7–10]. Moreover, the scope and scale of collecting HRSNs on patients are immense and costly [11]. While no optimal frequency for HRSN has been established [12], annual or even universal screening is a common recommendation [4] and the agency’s new quality reporting metrics require HRSN screening during every admission [13]. Such reporting expectations would be a sizable data collection effort for many organizations. Notably, the actual performance of many existing screening instruments to collect HRSNs is unknown [14] or even potentially poor [15].

Machine learning (ML) predictive models may be an alternative that addresses many of the practical challenges of systematically identifying patients with HRSNs who need services and resources. The predictive modeling process can be automated, eliminating time constraints, workflow issues, or staff availability that often impede the current collection and use of HRSN data. In addition, automated predictive modeling operates as a universal screening program: it is not subject to intentional or unintentional biases that lead individuals to selectively administer questionnaires [16] or to missing data due to patient nonresponse. In addition, ML techniques can capitalize on the ever-growing amount of longitudinal electronic health records (EHR), health information exchange (HIE), and non-healthcare organization data reflecting patient social circumstances and factors [17,18]. These data can provide a longitudinal and comprehensive view of the patient and are not dependent upon a single organization for data collection. ML predictive modeling has already demonstrated promise in identifying patients with HRSNs [19–21]. Nevertheless, the performance of ML predictive models has not yet been compared to other methods of identifying patients needing HRSN services.

## Objective

This study’s objective was to compare the predictive power of EHR features versus screening questionnaire features in identifying HRSNs in identifying emergency department (ED) patients needing services to address HRSNs. The ED is an appropriate setting for such a study. First, HRSNs are highly prevalent among ED patients [10]. Second, patient HRSNs drive adverse outcomes such as repeat ED visits [22] and additional ED services [23]. Relatedly, patients’ HRSNs complicate care delivery, inhibit treatment adherence, and act as a barrier to follow-up with primary and specialty care [22,24]. Third, the ED is an essential source of care for minoritized and under-resourced patient populations [25]. This is important because prior ML predictive models have been biased against these populations [26].

This analysis focuses on predicting a patient’s likely need for HRSN services and not identifying the actual presence of individual HRSNs. The rationale for this choice is to focus on the most actionable outcome for providers. Specifically, at the individual patient level, screening for HRSNs is primarily used to inform clinicians about the need for appropriate services [4,5,27]–most often to initiate a referral to social service professionals (e.g., social workers, case managers, and navigators) or community-based social service organizations best equipped to address HRSNs [5,28]. This is true regardless of the method of identification used (ML predictive modeling or questionnaire). Moreover, the difference between the presence of a HRSN and the need for HRSN services is of practical importance for health care organizations. Not all patients responding to HRSNs screening want or receive services [6,29,30]. By focusing our prediction modeling on those with a likely need for HRSN services, we are more directly targeting the resources and activities that healthcare providers and organizations will need to supply.

## Methods

**Fig 1:**
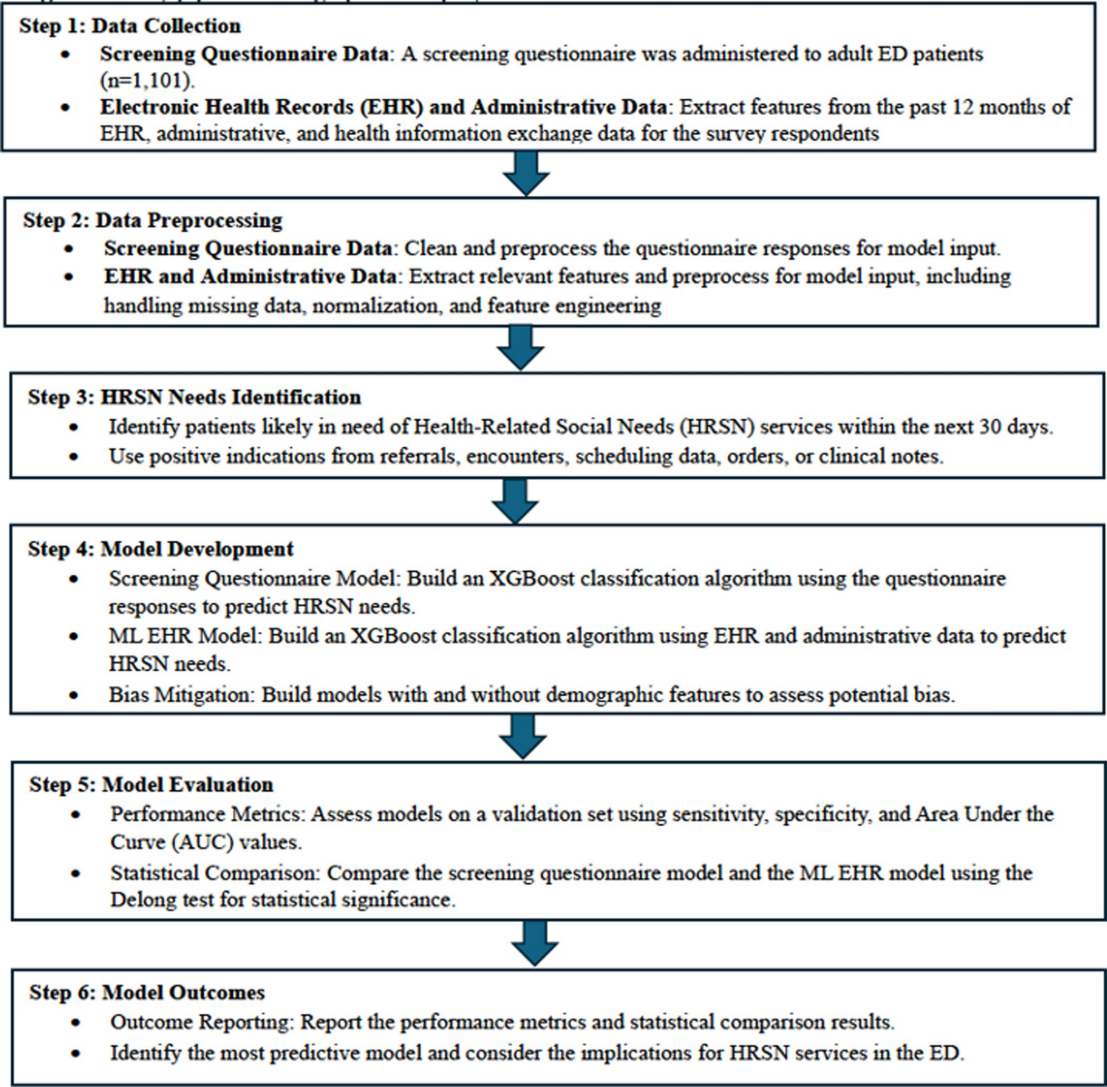
Study pipeline. The graphical display of the data elements and model outcomes.

Below, we describe the process of comparing two approaches to predict the likely need for an HRSN service (see the study pipeline in Fig 1). We built XGBoost classification algorithms using responses from a screening questionnaire (screening questionnaire model). Additionally, we built XGBoost classification algorithms using existing EHR, administrative, and health information exchange data (ML EHR model).

### Setting and sample

The study sample included 1,101 adult (18 years old) patients who sought care at the Eskenazi Health ED in Indianapolis, IN, between June 2021 and March 2023. Eskenazi Health is the community’s safety-net provider. Patients were ineligible for study inclusion if: 1) age <18 years old; 2) lack of decision capacity (e.g., psychosis, altered mental state, dementia) or 3) critical illness/injury impairing the patient’s ability to consent/participate. It is the policy of Indiana University that all research involving human subjects shall be subject to review or granted exemption by an appropriate Indiana University Institutional Review Board or designee before project initiation and without respect to funding or the source of funding. The Indiana University institutional review board approved all study procedures before the study started, and written informed consent was collected by all subjects.

### Data sources

#### Screening questionnaire

We collected primary data on patients’ HRSNs using a screening questionnaire administered during an ED encounter between June 2021 and March 2023. The questionnaire contained the Epic EHR’s social determinants screening module and social worker assessment forms questions, such as housing instability, food insecurity, transportation barriers, legal issues, and financial strain [31–35]. Trained data collection staff recruited patients to complete the questionnaire during their ED encounter. We also included an invitation to complete the questionnaire online within the discharge paperwork. Data collection was offered in both English and Spanish. Subjects self-reported their HRSNs by completing the questionnaire in REDCap [36,37]. Subjects provided written informed consent to link their responses to existing EHR, administrative, and health information exchange data. We created binary indicators of the presence or absence of each HRSN [31–35].

#### EHR and other clinical data sources

Eskenazi Health’s EHR was the primary source of demographic, clinical, encounter, and billing history data. For each patient completing the survey, we extracted the past 12 months of encounter types, procedure codes, orders, diagnoses, prescriptions, payer history, address history, contact information, age, and gender. Using all patients’ clinical documents and notes from the past 12 months, we applied our previously validated natural language processing (NLP) algorithms to identify housing instability, financial strain, history of legal problems, food insecurity, transportation barriers, and unemployment [38,39]. We also linked historical billing and payment data.

In addition, we linked responses to the data available for each respondent in the Indiana Network for Patient Care (INPC). The INPC is one of the largest and oldest multi-institutional clinical repositories [40], with over 13 billion data elements on 15 million patients from more than 117 different hospitals and 18,000 practices across the state. These data provided additional information on patient encounters and diagnoses outside Eskenazi Health. Lastly, using a patient address, we linked to the most recent area deprivation index scores [41]. The transformation of these data sources into specific features is detailed in Features for EHR models, below. All data linkage was completed between April 2023 and December 2023. The authors had no access to information that could identify individual participants during or after data collection.

### Prediction target

We defined our prediction as the patient’s likely need for an HRSN service within the next 30 days of survey completion. Patients who met this definition were identified by a positive indication from the following data sources: referrals, encounters, scheduling data, orders, or clinical notes [21,42–44]. Eskenazi Health offers social workers, financial counseling, or medical/legal partnerships “in-house” through referrals. A positive indicator of a likely HRSN needs to include any referral to these services (whether resulting in an appointment or not), any encounter with these services (whether scheduled, kept, missed, or “no-show”), or any order mentioning these services or HRSNs. Order text or clinical notes that mentioned referrals to, or the need for, social workers, case managers, financial counselors, or case conferences were identified through keyword searches [42]. Data collection staff and study team members did not share results from the questionnaires with clinicians, so any decisions or actions did not reflect patients’ answers to study data collection.

### Features for ML EHR models

Following our prior work, we extracted and engineered over 100 features that could indicate a patient with a likely need for HRSNs services [45]. The individual patient features were reflected in the following categories. *Demographics and contact information* included features derived from patient’s addresses or contact information. *Encounter history* included all features representing visit types and frequencies. The *Clinical* class included all features resulting from diagnoses, documentation, or care delivery processes. The *HRSN screeners* class included all prior social needs screening results. The *Text* class included the indicators extracted via NLP. The *Geospatial* class included the area deprivation index. The *Financial* class included features derived from billing, payment, and insurance data. All measures were for the 12 months before the completion of the screening instrument. Throughout the paper, we collectively refer to all these features as the EHR-based data and detail in S1 File.

### Modeling and comparative analyses

To develop the ML EHR models, we built ML classifiers using XGBoost [46], an ensemble-based classification algorithm that employs gradient boosting to add decision trees to address errors in prior predictions, resulting in a robust decision model. Models were developed in Python using 5-fold cross-validation and a grid search for hyperparameter tuning. To support interpretation, we extracted XGBoost’s feature importance scores (based on F scores) and utilized the SHAP (SHapley Additive exPlanations) method to summarize the contributions of features to the models [47]. Models were assessed on the validation set using sensitivity, specificity, and Area Under the Curve (AUC) values. The HRSN screening questions were not included in the ML EHR models.

For the screening question models, we also built an XGBoost classification algorithm. The input features were the binary indicator of a positive screen for each of the 5 assessed HRSN. We followed the same modeling approach as above and calculated sensitivity, specificity, and AUC values. We adopted the approach purposefully. For one, screening questionnaires with multiple HRSNs do not include guidelines for creating an overall risk score [48]. As our prediction target includes services that potentially reflect multiple HRSNs or professionals capable of addressing various HRSNs, we required the screening questions to produce a single prediction result. Additionally, we wanted a consistent comparison between the screening question models and the ML EHR models; using alternative classification methodologies would have introduced potential alternative explanations for performance differences.

We recognize that ML modeling of healthcare data may demonstrate or perpetuate biases against certain populations [26] and that differences in access to care or other structural barriers could influence the distribution of our outcome, screening questionnaire responses, or measures of prior utilization by demographics (S2 File). Therefore, we took several steps in the analysis to mitigate bias. First, we ran all models with and without age and gender to identify potential differences for these groups of patients. Second, race and ethnicity have been used with uncertain purpose in past algorithms [49]. Therefore, we did not include race or ethnicity as input features in any model. Instead, we stratified all models by patient self-reported race and ethnicity as a check on model fairness (i.e., consistent performance).

We described the sample using frequencies and percentages. We used the equality of proportions test to compare AUC values using the Delong test and the difference in sensitivity, specificity, and PPV [50].

## Results

The study sample was predominately female (58.9%) and reflective of an urban, safety-net ED population, with less than a third of the sample being non-Hispanic White (31.5%). The HRSNs were common, with half of the patients reporting food insecurity (46.0%) and housing instability (46.0%) (Table 1). Almost half (41%) of the patients had a positive indicator for the likely HRSN service needs within the next 30 days, as identified through referrals, encounters, scheduling data, orders, or clinical notes (Table 1).

**Table 1:** Adult emergency department patients by indication of health-related social need (HRSN) service need, Indianapolis, IN.

	Total	No HRSN service	HRSN service need in next 30 days	p-value
	n=1,101	n=650	n=451	
Age (mean, sd)	41.5 (15.1)	38.7 (14.5)	45.6 (15.1)	<0.001
Female gender	649 (58.9%)	397 (61.1%)	252 (55.9%)	0.084
Race & ethnicity				0.003
Asian	11 (1.0%)	8 (1.2%)	3 (0.7%)	
Black non-Hispanic	511 (46.4%)	322 (49.5%)	189 (41.9%)	
Hispanic	203 (18.4%)	116 (17.8%)	87 (19.3%)	
Multiple	14 (1.3%)	7 (1.1%)	7 (1.6%)	
Other / unknown	15 (1.4%)	14 (2.2%)	1 (0.2%)	
White non-Hispanic	347 (31.5%)	183 (28.2%)	164 (36.4%)	
Language other than English	129 (11.7%)	68 (10.5%)	61 (13.5%)	0.12
Encounter history^a^				
Inpatient admissions (mean, sd)	0.8 (3.0)	0.5 (1.7)	1.3 (4.2)	<0.001
Emergency department visits (mean, sd)	5.2 (14.0)	4.2 (9.2)	6.5 (18.9)	0.007
Primary care visits (mean, sd)	3.3 (5.4)	2.8 (4.6)	4.0 (6.4)	<0.001
Elixhauser co-morbidity score (mean, sd)	1.5 (1.9)	1.0 (1.3)	2.3 (2.2)	<0.001
Health-related social needs^b^				
Financial strain	228 (20.7%)	114 (17.5%)	114 (25.3%)	0.002
Food insecurity	692 (62.9%)	408 (62.8%)	284 (63.0%)	0.95
Housing instability	506 (46.0%)	287 (44.2%)	219 (48.6%)	0.15
Transportation barriers	416 (37.8%)	215 (33.1%)	201 (44.6%)	<0.001
Legal problems	239 (21.7%)	128 (19.7%)	111 (24.6%)	0.052

^a^during 12 months prior to survey date.

^b^per Epic SDOH screening questions.

Patients with an indicator for the likely HRSN service needs in the next 30 days differed from those without on several characteristics. Patients with an indicator for the HRSN service needs were older, had more comorbidities, and had higher prior utilization. Additionally, the distribution of patients with and without an indicator for the HRSN service needs in the next 30 days differed by race and ethnicity (p = 0.003). Finally, patients with an indicator for the HRSN service needs had higher reported financial strain (25.3% vs. 17.5%; p = 0.002) and transportation barriers (44.6% vs. 33.1%; p<0.001).

### ML EHR data model

The model using the EHR-based data sources (without demographics) had an AUC = 0.765 (95%CI = 0.737, 0.792) (Table 2). The model was more specific (0.790; 95%CI = 0.753, 0.822) than sensitive (0.600; 95%CI = 0.560, 0.640), and the positive predictive value (PPV) was 0.756 (95%CI = 0.715, 0.793). Including patient age and gender in the models resulted in a small but not statistically significant increase in overall performance (AUC = 0.772; 95%CI = 0.744, 0.800; p = 0.5000).

**Table 2:** Comparison of models using screening questionnaire and EHR data in predicting need for health-related social service within 30 days of emergency department visit.

	ML EHR model	ML EHR model with age & gender	Screening questionnaire model	Screening questionnaire model with age & gender
AUC	0.765 (0.737, 0.792)	0.772 (0.744, 0.800)	0.580 (0.546, 0.611)	0.640 (0.609, 0.672)
Sensitivity	0.600 (0.560, 0.640)	0.646 (0.606, 0.684)	0.578 (0.537, 0.617)	0.613 (0.572, 0.652)
Specificity	0.790 (0.753, 0.822)	0.765 (0.727, 0.799)	0.566 (0.524, 0.608)	0.583 (0.541, 0.625)
PPV	0.756 (0.715, 0.793)	0.749 (0.709, 0.785)	0.591 (0.550, 0.631)	0.615 (0.574, 0.654)

AUC = area under the curve.

PPV = positive predictive value.

#### Screening questionnaire model

The screening questionnaire model performed worse than the ML HER model (Table 2). Using only the screening questions, the AUC value of 0.580 (95%CI = 0.546, 0.611) was lower than the ML model (p < 0.0001). Additionally, while sensitivity was not statistically different between the two models, the specificity of the screening questionnaire model (0.566; 95%CI = 0.524, 0.608) was lower than the ML EHR model (p<0.0001). The positive predictive value for the screening questionnaire model was 0.591 (95%CI = 0.550, 0.631), which was also lower than the ML EHR model (p<0.0001). Adding patient age and gender to the screening questionnaire model resulted in slight improvements in overall performance (AUC = 0.640; 95%CI = 0.609, 0.672; p = 0.0117). However, this model still had lower performance than the ML EHR model with demographics (p < 0.0001).

**Fig 2:**
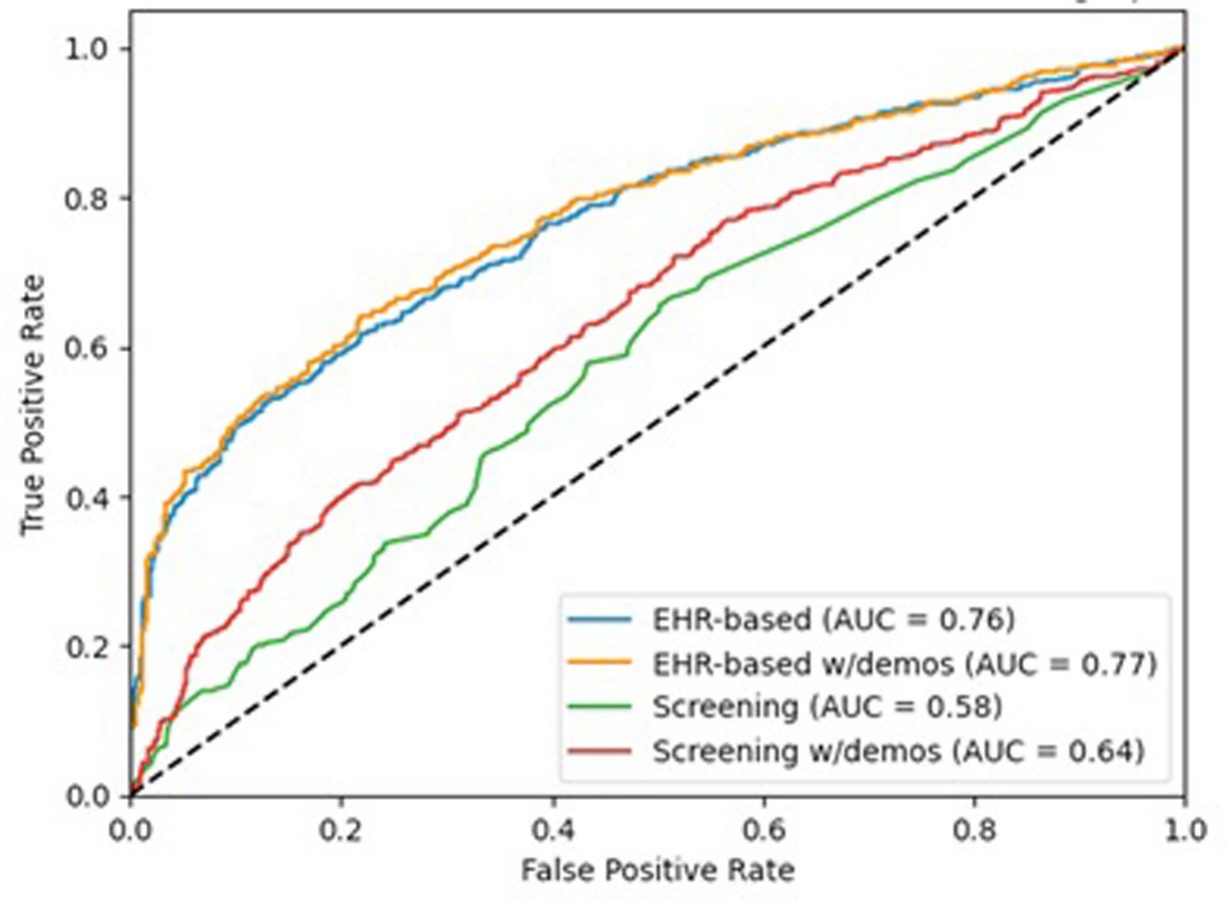
ROC Curves of EHR and screening question models predicting the need for HRSN service within 30 days of an emergency department visit, with and without age and gender.

**Fig 3:**
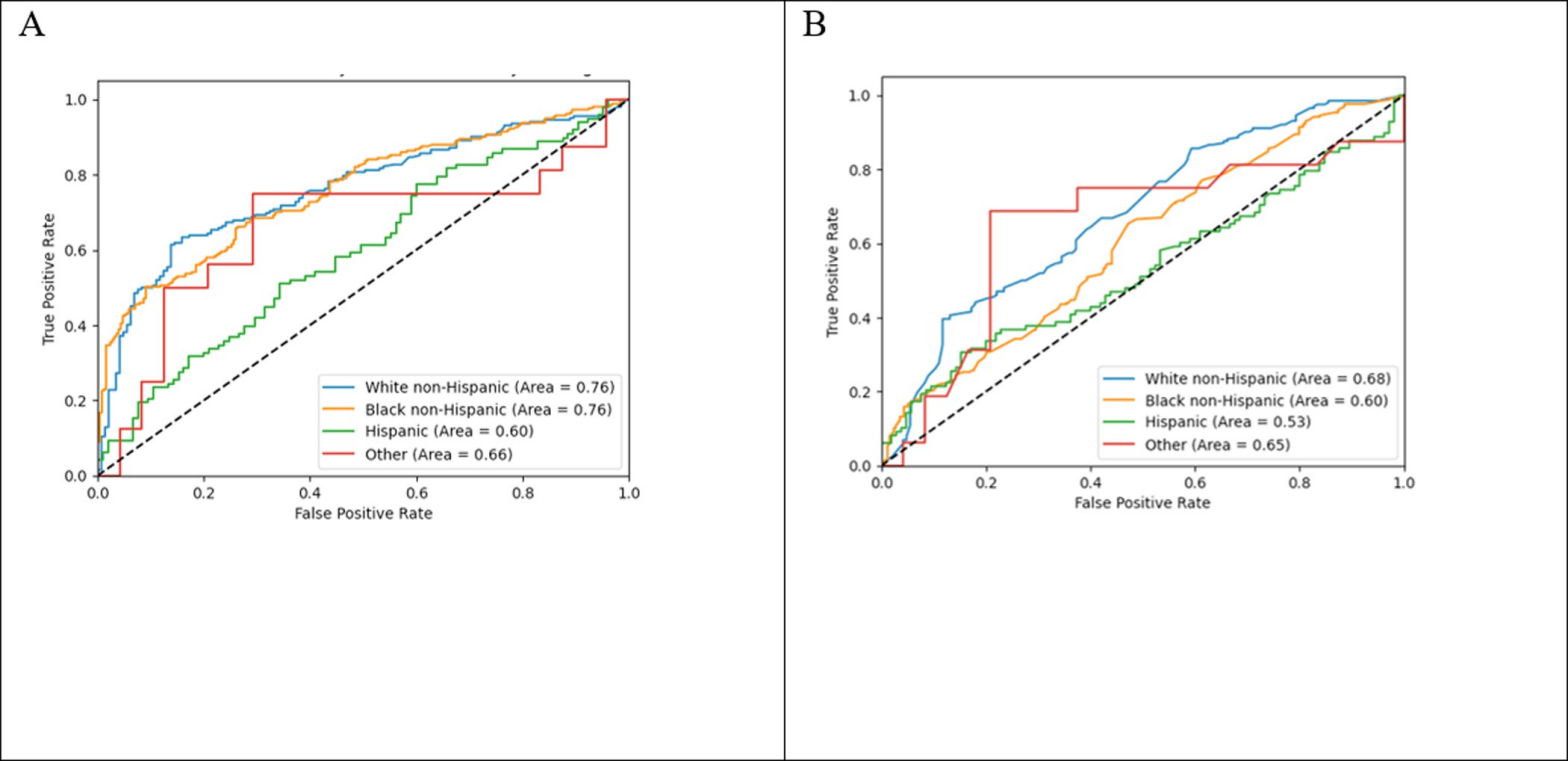
ROC Curves of (A) EHR-based and (B) screening question models predicting need for HRSN service within 30 days of emergency department visit by race and ethnicity.

The differential performance is illustrated in Fig 2, with the largest ROC curves reflecting the ML predictive models. For the ML predictive models without controlling for demographics (S3 File Panel A), the five most important features in predicting an indication for the HRSN need for services were the patient’s Elixhauser comorbidity score, a prior housing instability ICD-10 Z code, number of different therapeutic classes prescribed, legal problems documented in clinical notes, and total primary care visits. Including patient age and gender in the ML predictive models resulted in the same top five features, with some variation in ordering (S3 File Panel B). Notably, age was the sixth most important feature in the model with demographics, outranking other direct measures of HRSN. The most important feature of the screening questionnaire model was the transportation barrier (S3 File Panel C). Including demographics in the screening questionnaire model made age the most important feature (Fig 3 Panel D).

#### Stratification by race and ethnicity

The ML predictive and screening questionnaire models stratified by race and ethnicity varied in their overall performance (Fig 3). The stratified AUCs varied significantly by the patient’s race and ethnicity for the ML predictive models (p = 0.0241) and the screening questionnaire model (p = 0.0392). Differences by race and ethnicity persisted even if the minor " *other* " group was omitted from the analyses. Beyond overall performance, notable differences existed in other performance measures (Table 3).

**Table 3:** Comparison of models using screening questionnaire and EHR data in predicting need for health-related social service within 30 days of emergency department visit stratified by self-reported race and ethnicity.

	ML EHR model				Screening questionnaire model			
	White Non-Hispanic	Black Non-Hispanic	Hispanic	Other Non-Hispanic	White Non-Hispanic	Black Non-Hispanic	Hispanic	Other Non-Hispanic
	n=347	n=511	n=203	n=40	n=347	n=511	n=203	n=40
AUC	0.766	0.753	0.601	0.577	0.659	0.536	0.454	0.404
Sensitivity	0.703	0.591	0.357	0.188	0.678	0.370	0.276	0.063
Specificity	0.697	0.748	0.676	0.875	0.545	0.756	0.657	0.875
PPV	0.763	0.704	0.507	0.500	0.675	0.605	0.429	0.250

In both the ML predictive and screening questionnaire models, the sensitivity was roughly equal to or higher than the specificity for White, non-Hispanic patients. However, for all other respondents, both models had higher specificity than sensitivity. In other words, both models were better at finding White, non-Hispanic patients with HRSN service needs than it was at finding patients from other races and ethnicities with HRSN service needs. Additionally, which features were most important changed when each model was stratified by race and ethnicity (S3 File).

## Discussion

In predicting which adult ED patients would need HRSN services within the next 30 days, ML predictive models leveraging several robust EHR data sources outperformed models using screening questions only. While comparatively better, the ML predictive models using EHR data sources did not exhibit particularly strong performance, and all models demonstrated bias.

Collecting HRSN information is a growing necessity for healthcare organizations due to quality reporting requirements as part of CMS’ Hospital Inpatient Quality Reporting (IQR) program [51] and the Merit-based Incentive Payment System (MIPS) [52], new National Committee on Quality Assurance (NCQA) HEDIS quality measures [53], and Joint Commission accreditation requirements [54]. The collection of HRSN data can be used to drive individual patient referrals, help inform providers’ decision-making processes, and support organizational quality improvement and measurement efforts [27]. However, our findings indicate that models using the HRSN screening questions were poor predictors of future HRSN service needs. Indeed, these models were only slightly better than a coin flip. The low performance of models using screening questions could stem from the overwhelming number of positive results, making it hard to identify who requires services in a universally high-risk group. This criticism is not unique to HRSN questionnaires; although ML predictive models using EHR data sources performed better, they failed to reach accepted thresholds for clinical usefulness [55]. Nevertheless, the limited ability of the HRSN information to predict who needs HRSN services can pose a challenge, as HRSNs are often highly prevalent among patients at many healthcare organizations [10,56].

The widely utilized general HRSNs screening questionnaires, such as PRAPARE, Epic, HealthLeads or CMS’s Accountable Health Communities tool, do not account for demographics. Although, PRAPARE’s tally method of scoring for overall social risk includes race and ethnicity as contributing factors [57]. However, in this study, adding gender and age led to minor improvements in the performance of the ML EHR model and significant improvements in the screening questionnaire model. HRSNs vary across patient age groups [58], and when added to the screening question models, age was the most important feature. Thus, one potential improvement to HRSN screening questions could be incorporating age. Some domain-specific screening instruments, like the Consumer Financial Protection Bureau’s Financial Well-Being Scale [59], account for differences between age groups. Additionally, women are likely to experience poverty at higher rates than men [58] but adding gender was not as important in the models. A potential path for future work could be the appropriate and equitable integration of age and other demographics into screening question interpretation.

Overall, the ML EHR model performed better than the screening questionnaire model. The ML EHR modeling approach has the obvious advantage of drawing upon more information and, thus, is an increasingly preferred approach to HRSN measurement [19–21]. While model performance still requires significant improvement, our findings highlight the potential value of several EHR data elements already accessible to healthcare organizations. Prior ICD-10 Z-codes and HRSN documented in clinical notes were important model features. As healthcare organizations already collect and maintain the HRSN data, it can be readily leveraged to support existing HRSN measurement activities as potential means of imputing missing responses or by driving decision support systems on patients for additional HRSN screening.

Problematically, the ML EHR and the screening questionnaire models exhibited differential performance and bias across patient race and ethnicity. Biased models perpetuate health inequities [59,60]. The bias observed in our models is likely due to both measurement bias and representation bias [61]. Measurement bias can affect the performance of the ML predictive and screening questionnaire models. For example, while data points like ICD-10 Z codes are potentially informative, they are often underutilized and inconsistent [62]. Even though the screening questions are not perfect instruments, they have psychometric properties that vary according to specific HRSNs [15]. Representation bias may be more influential for the ML EHR models, as many of the most important features were correlated with access to care and services. To have a higher Elixhauser score or more medication classes or prior diagnoses requires access to the healthcare system for diagnoses and services. This is important because access to care is not equivalent across populations [63]. Although we recruited a diverse sample, that may not account for potential differences in the patients’ underlying data due to differential experiences with the healthcare system.

This study reinforces the need for a detailed examination of the differential performance of risk prediction models. Simply focusing on the differential overall performance could miss the actual effects such models can have in practice. Of particular concern is the fact that the observed bias predominately affected model sensitivity, which is the proportion of true positives that are correctly identified as positive [64]. As such, the models, including those using the screening questions, would under-identify members of certain race and ethnic groups needing HRSN services. If applied to care practices, under-identification would run contrary to patients’, physicians’, staff members’, and organizations’ expectations for HRSN measurement and intervention activities [2,65]. Such differential under-identification would likely perpetuate health inequities. Obviously, before implementing practice, models must be checked for bias. In addition, the effectiveness and impact of any bias mitigation strategies requires evaluation [66].

### Limitations

This study faces several limitations, particularly in terms of external validity. First, our models predict a future event–the likely need for HRSN services. However, the availability of the data, the nature of the services, and workflow processes unique to the setting may limit the generalizability of the outcome to other settings. Our models may not be generalizable either. We can draw on a large set of EHR and health information exchange data, including financial information and data extracted via NLP. Likewise, our choice of screening questions may differ from other available screening tools. Additionally, our findings may not hold over time as screening practices tend to degrade [7] and prediction models drift [67] over time.

Lastly, HRSN screening via questionnaires aims to drive referrals to services to address patient needs [4,68]. However, our choice of outcome measure may have limited the potential predictive ability of our models. It is possible the screening questions better capture the concept of social “risk” [69], that is, the presence of a potentially adverse factor to health. By contrast, the predicted outcome may have better reflected the concept of social “need”, that is, an immediate concern that aligns with patient’s priorities and preferences [69,70]. These are related, but distinct, concepts.

## Conclusions

The ML predictive model performed better than the screening questionnaire model, yet both models demonstrated biases. Additional work is needed to design predictive models that effectively identify all patients with HRSNs.

## Supporting information

S1 File Engineered features from electronic health record (EHR) and health information exchange (HIE) data (past 12 months). (DOCX)

S2 File Comparison of features. (DOCX)

S3 File Feature importance values by race and ethnicity. (DOCX)
