# Supplementary material for: Comparing the performance of screening surveys versus predictive models in identifying patients in need of health-related social need services in the emergency department
Source: PLoS One. 2024 Nov 20;19(11):e0312193. doi: 10.1371/journal.pone.0312193 (PMC11578524; doi:10.1371/journal.pone.0312193)
Supplement: S1 File — (DOCX) [file pone.0312193.s001.docx]

Supporting File 1. Engineered features from EHR and HIE data (past 12 months)

| Demographics & contact |  |
| --- | --- |
| address_change | Number of changes in addresses (implement at census block level) within 12 months of survey date |
| phone_change | Number of changes in phone numbers |
| no_phone | No phone number |
| address_hosp | Address is the same as the hospital / ED |
| address_homeless | Address is known alias for homelessness or addresses linked to known shelter addresses |
| address_legal | Address is listed as prison / law enforcement^5^ |
| phone_shelter | Phone number a known homeless shelter^1^ |
| phone_legal | Phone number a known correction / jail |
| marital_single  marital_sepdev  marital_widow  marital_othunk | Marital Status |
| marital_change | Changes to marital status |
| name_change | Number of different names used in EHR^7^ |
| no_emergency | No emergency contact |
| occup_unemp | listed as Unemployed |
| employ_part  employ_emp  employ_outofworkforce  employ_unk | Employment status |
| no_email | No email on file |
| phr | active EHR portal account |
| no_ssn | No social security number on file |
| Financial |  |
| insurance_2public | Change in insurance type from commercial to public/ medicaid / dual / self-pay |
| insurance_2dual | Change in insurance type from Medicare to dual status |
| insurance_uninsured | Uninsured or self-pay status |
| insurance_public | Payer was a means tested program (Medicaid, Dual, other public) |
| insurance_legal | Payer was justice system / law enforcement / corrections |
| fin_nopaymenthosp | Had encounter where no payments were collected (total charge = total adjustments) hospital part |
| fin_balancehosp | Had account balance hospital part |
| fin_collectedlesshosp | Collected less than total balance hospital part |
| fin_cashpayhosp | Payment by cash hospital part |
| fin_nopaymentpb | Had encounter where no payments were collected (total charge = total adjustments) physician part |
| fin_balancepb | Had account balance physician part |
| fin_collectedlesspb | Collected less than total balance physician part |
| fin_cashpaypb | Payment by cash physician part |
| fin_legal | Payment source was law enforcement |
| Encounters |  |
| enc_miss_add | % of encounters with missing address information |
| enc_noshow | % of outpatient visits that were no shows |
| enc_cancel | % of outpatient visits that were canceled |
| enc_lama | % of outpatient visits that were left without being seen |
| enc_totpc | Total number of primary care visits |
| enc_toted | Total number of ED visits |
| enc_totbh | Total number of behavioral health visits |
| enc_totip | Total number of Inpatient admissions |
| enc_fincoun | Prior visit with financial counseling (regardless of keep, cancelled, completed) |
| enc_sw | Prior visit with social worker (regardless of keep, cancelled, completed) |
| enc_mlp | Prior visit with medical legal partnership(regardless of keep, cancelled, completed) |
| enc_ed2out | Ratio of emergency department to office visits |
| enc_arrival_legal | Mode of arrival related to law enforcement |
| enc_admission_legal | Source of admission was related to law enforcement / legal system |
| enc_dispo_legal | Disposition location was related to law enforcement / legal system |
| enc_dispo_legal_inpc | Disposition location was related to law enforcement / legal system INPC |
| enc_arrival_taxi | Mode of arrival was taxi or public transport |
| Clinical |  |
| icd_crime | Prior *ICD* codes for injuries related to crimes (eg, firearms, stabbings): |
| icdz_housing | Prior Housing insecurity ICD10 Z codes |
| icdz_financial | Prior Financial strain ICD10 Z codes: |
| icdz_transport | Prior Transportation barriers ICD10 Z codes |
| icdz_food | Prior Food insecurity ICD10 Z codes: |
| icd_food | Prior Food insecurity ICD10 codes: |
| icdz_unemploy | Prior Unemployment ICD10 Z codes: |
| icd_legal | Prior Legal problems ICD10 codes (prison place of event & legal intervention) or Z codes |
| elixhauser | Prior Elixhauser comorbidity index score |
| icd_lead | Prior ICD10 code for lead poisoning |
| ref_sw | Prior Referral to social work |
| ref_financial | Prior Referral to financial counseling |
| ref_mlp | Prior Referral to medical legal partnership |
| ref_food | Prior Referrals / Orders to WIC, Meals on Wheels, Cafeteria Vouchers, SNAP/TANF, food stamps, food pantries |
| ref_housing | Prior Patient directed to resources to address housing instability |
| ref_transportation | Prior Patient directed to resources to address transportation barriers |
| ordrtxt_housing | Prior Orders mention homelessness or housing^2^ |
| ordrtxt _food | Prior Orders mention food or hunger^2^ |
| ordrtxt _legal | Prior Orders mention legal issues or incarceration issues^2^ |
| ordrtxt _financial | Prior Orders mention financial^2^ |
| ordrtxt _unemploy | Prior Orders mention unemployment^2^ |
| ordrtxt _transport | Prior Orders mention transportation^2^ |
| ordrtxt _sw | Prior Orders mention social work^2^ |
| order_lead | Prior Order for lead screening |
| rx_classes | Prior Total number of different therapeutic classes prescribed |
| tobacco | Tobacco usage (current) |
| phq9_dep | Prior Positive depression screen |
| phq9_anx | Prior Positive anxiety screen |
| HRSN screeners |  |
| sw_homeless | Prior Social worker assessment identified homelessness |
| sw_food | Prior Social worker assessment identified food insecurity |
| sw_legal | Prior Social worker assessment identified incarceration history |
| sw_financial | Prior Social worker assessment identified, financial strain or no income |
| sw_uenmploy | Prior Social worker assessment identified unemployment |
| sw_transport | Prior Social worker assessment identified transportation barriers |
| screen_housing | Prior EHR screener identified housing instability |
| screen_food | Prior EHR screener identified food insecurity |
| dv_screen_financial | DV EHR screener identified, financial strain(within next 30 days inclusive of visit) |
| screen_transport | Prior EHR screener identified transportation barriers |
| Geospatial |  |
| area_moved2lower | Moved from higher income census tract to lower income census tract |
| area_adi | Area deprivation index (ADI) state ranking (census block group) |
| Text |  |
| nlp_housing | Prior Any mention of housing |
| nlp_financial | Prior Any mention of financial strain |
| nlp_unemploy | Prior Any mention of unemployment |
| nlp_tranport | Prior Any mention of transportation |
| nlp_legal | Prior Any mention of legal issues |
| nlp_food | Prior Any mention of food insecurity |
|  | Features not used in modeling |
| age | Age in years |
| race_ethnicity | Race/ethnicity OMB as categorical |
| Female | Gender (male, female, non-binary/other) |
| Language_ne_english | Preferred language not English |
| interpreter | Translations services used / Interpreter needed |
