# Supplementary material for: Comparing the performance of screening surveys versus predictive models in identifying patients in need of health-related social need services in the emergency department
Source: PLoS One. 2024 Nov 20;19(11):e0312193. doi: 10.1371/journal.pone.0312193 (PMC11578524; doi:10.1371/journal.pone.0312193)
Supplement: S2 File — (DOCX) [file pone.0312193.s002.docx]

**Supporting File 2**

Table A1. Comparison of features for White non-Hispanic respondents and all other non-White non-Hispanic respondents.

+-----------------------------------------------------------------------------+

| Total Non-WNH White-NH p-value |

|-----------------------------------------------------------------------------|

| N=1,101 N=754 N=347 |

|-----------------------------------------------------------------------------|

| epic_financestrain 228 (20.7%) 143 (19.0%) 85 (24.5%) 0.035 |

|-----------------------------------------------------------------------------|

| epic_foodinsecure 692 (62.9%) 479 (63.5%) 213 (61.4%) 0.49 |

|-----------------------------------------------------------------------------|

| epic_housinginstability 506 (46.0%) 345 (45.8%) 161 (46.4%) 0.84 |

|-----------------------------------------------------------------------------|

| epic_transport 416 (37.8%) 287 (38.1%) 129 (37.2%) 0.78 |

|-----------------------------------------------------------------------------|

| epic_legal 239 (21.7%) 145 (19.2%) 94 (27.1%) 0.003 |

|-----------------------------------------------------------------------------|

| enc_totip_inpc 0.8 (3.0) 0.6 (2.9) 1.1 (3.3) 0.010 |

|-----------------------------------------------------------------------------|

| enc_toted_inpc 5.2 (14.0) 4.2 (12.1) 7.2 (17.4) 0.001 |

|-----------------------------------------------------------------------------|

| enc_totpc 3.3 (5.4) 3.4 (5.2) 3.0 (5.8) 0.20 |

|-----------------------------------------------------------------------------|

| elixhauser_inpc 1.5 (1.9) 1.5 (1.8) 1.7 (2.0) 0.048 |

+-----------------------------------------------------------------------------+

Data are presented as mean (SD) for continuous measures, and n (%) for categorical measures.

Table A2. Comparison of features for female respondents and all other respondents.

+-----------------------------------------------------------------------------+

| Total Others Female p-value |

|-----------------------------------------------------------------------------|

| N=1,101 N=452 N=649 |

|-----------------------------------------------------------------------------|

| epic_financestrain 228 (20.7%) 99 (21.9%) 129 (19.9%) 0.41 |

|-----------------------------------------------------------------------------|

| epic_foodinsecure 692 (62.9%) 269 (59.5%) 423 (65.2%) 0.056 |

|-----------------------------------------------------------------------------|

| epic_housinginstability 506 (46.0%) 219 (48.5%) 287 (44.2%) 0.17 |

|-----------------------------------------------------------------------------|

| epic_transport 416 (37.8%) 173 (38.3%) 243 (37.4%) 0.78 |

|-----------------------------------------------------------------------------|

| epic_legal 239 (21.7%) 149 (33.0%) 90 (13.9%) <0.001 |

|-----------------------------------------------------------------------------|

| Language_ne_english 129 (11.7%) 48 (10.6%) 81 (12.5%) 0.34 |

|-----------------------------------------------------------------------------|

| enc_totip_inpc 0.8 (3.0) 0.6 (2.7) 0.9 (3.3) 0.048 |

|-----------------------------------------------------------------------------|

| enc_toted_inpc 5.2 (14.0) 4.0 (13.8) 6.0 (14.1) 0.021 |

|-----------------------------------------------------------------------------|

| enc_totpc 3.3 (5.4) 2.5 (4.2) 3.9 (6.1) <0.001 |

|-----------------------------------------------------------------------------|

| elixhauser_inpc 1.5 (1.9) 1.5 (1.9) 1.6 (1.9) 0.48 |

+-----------------------------------------------------------------------------+

Data are presented as mean (SD) for continuous measures, and n (%) for categorical measures.
