# Supplementary material for: Comparing the performance of screening surveys versus predictive models in identifying patients in need of health-related social need services in the emergency department
Source: PLoS One. 2024 Nov 20;19(11):e0312193. doi: 10.1371/journal.pone.0312193 (PMC11578524; doi:10.1371/journal.pone.0312193)
Supplement: S3 File — (DOCX) [file pone.0312193.s003.docx]

Appendix 3. Feature importance values from EHR-based and screening question models.

| A. EHR-based model | B. EHR-based model with demographics |
| --- | --- |
| 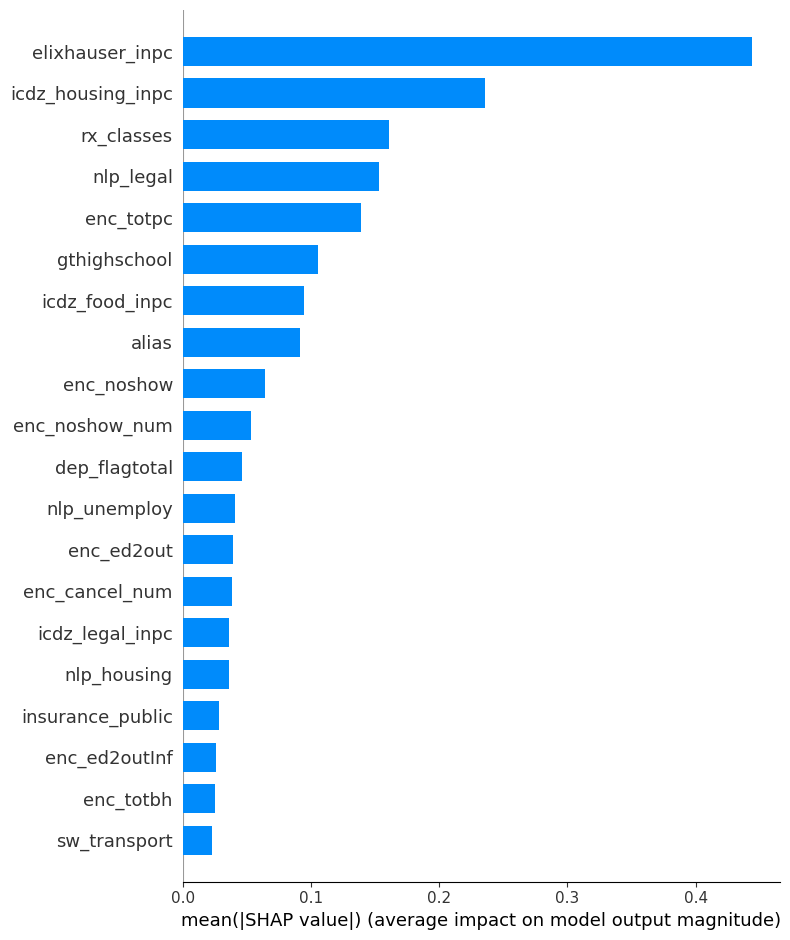 | 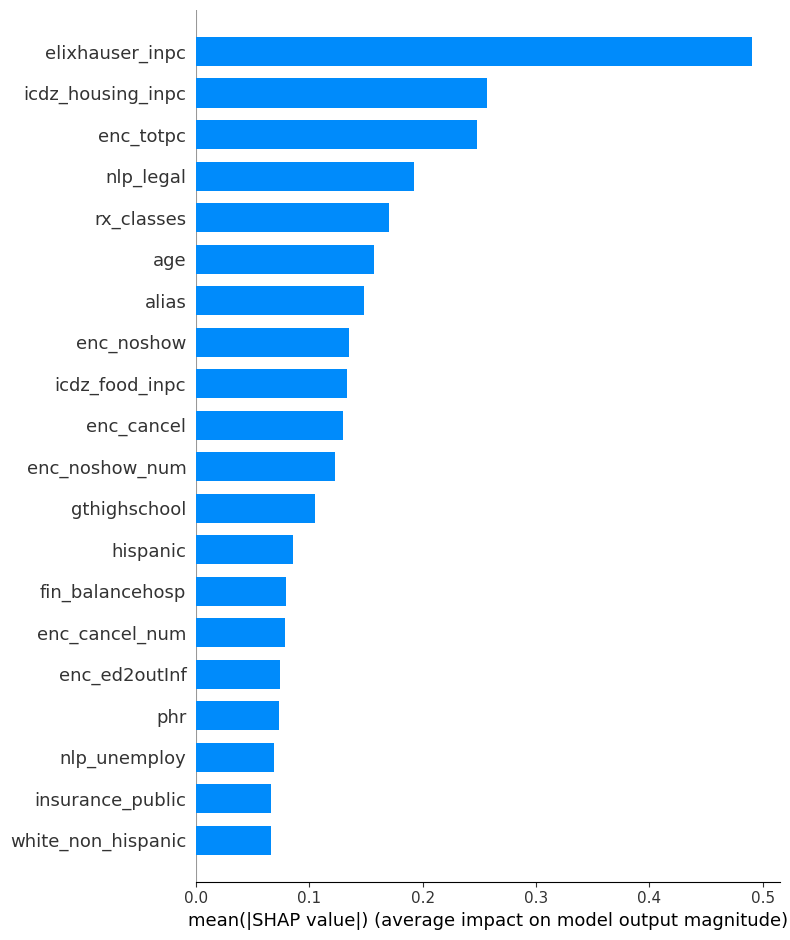 |

| C. Screening questions model | D. Screening questions model with demographics |
| --- | --- |
| 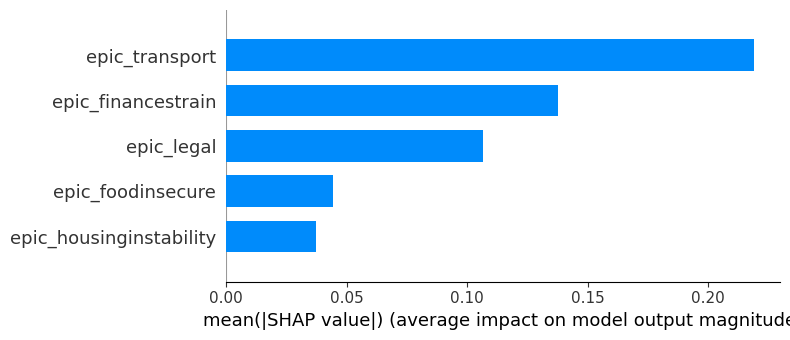 | 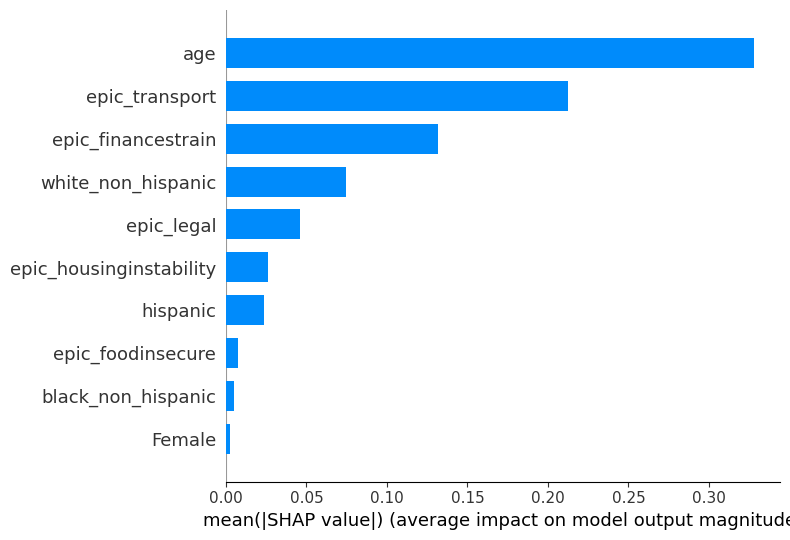 |
